# Transition of care of patients with chronic diseases and its relation with clinical and sociodemographic characteristics *

**DOI:** 10.1590/1518-8345.6594.4014

**Published:** 2023-10-09

**Authors:** Larissa Berghetti, Márcia Baiocchi Amaral Danielle, Vanessa Dalsasso Batista Winter, Ane Gabriele Poli Petersen, Elisiane Lorenzini, Adriane Cristina Bernat Kolankiewicz

**Affiliations:** 1 Universidade Regional do Noroeste do Estado do Rio Grande do Sul, Ijuí, RS, Brasil.; 2 Universidade Regional do Noroeste do Estado do Rio Grande do Sul, Curso de Enfermagem, Ijuí, RS, Brasil.; 3 Universidade Federal de Santa Catarina, Florianópolis, SC, Brasil.; 4 Becaria del Conselho Nacional de Desenvolvimento Científico e Tecnológico (CNPq), Brasil.

**Keywords:** Transitional Care, Patient Readmission, Chronic Diseases, Patient Discharge, Continuity of Patient Care, Patient-Centered Care, Cuidado de Transición, Readmisión del Paciente, Enfermedades Crónicas, Alta Hospitalaria, Continuidad de la Atención al Paciente, Atención Dirigida al Paciente, Cuidado Transicional, Readmissão do Paciente, Doenças Crônicas, Alta Hospitalar, Continuidade da Assistência ao Paciente, Assistência Centrada no Paciente

## Abstract

**Objective:**

evaluate the transition of care from the perspective of people living with chronic diseases and identify its relation with clinical and sociodemographic characteristics.

**Method:**

cross-sectional study with 487 patients who were discharged from a hospital. Clinical and sociodemographic characterization instruments were used, as well as the Care Transitions Measure-15, which measures Preparation for self-management, Secured preferences, Understanding about medications and Care plan factors. Descriptive and inferential statistical analysis.

**Results:**

the transition of care was satisfactory (76.8±10.4). Average of the factors: Preparation for self-management (82.2±10.8), Secured preferences (84.7±14.3), Understanding about medications (75.7±13.7) and Care plan (64.5±13.2). Female patients had a higher average in the understanding about medications factor. Whites and residents in the urban area better evaluated the Care plan factor. The highest mean was observed for the Secured preferences factor (84.7±14.3) and the lowest for the Care plan factor (64.5±13.2). In all factors, significant differences were found in the variables (surgical patient, carrying clinical artifacts and not being hospitalized for COVID-19). Patients hospitalized for up to five days showed statistical difference in Preparation for self-management and Understanding about medications factors. In patients who were not readmitted within 30 days of discharge, Preparation for self-management was better. The better the Preparation for self-management, the lower the 30-day readmission rates.

**Conclusion:**

in patients living with chronic diseases, sociodemographic and clinical variables are associated with the transition of care. Patients who better evaluated preparation for self-management had fewer readmissions within 30 days.

## Abstract (es)

**Objetivo:**

evaluar la transición del cuidado desde la perspectiva de las personas que viven con enfermedades crónicas e identificar su relación con las características clínicas y sociodemográficas.

**Método:**

estudio transversal, con 487 pacientes dados de alta de un hospital. Se utilizaron los instrumentos de caracterización clínica, sociodemográfica y *Care Transitions Measure-15*, que mide los factores Preparación para el automanejo, Preferencias aseguradas, Comprensión sobre medicamentos y Plan de cuidados. Análisis estadístico descriptivo e inferencial.

**Resultados:**

la transición del cuidado fue satisfactoria (76,8±10,4). Media de factores: Preparación para el automanejo (82,2±10,8), Preferencias aseguradas (84,7±14,3), Comprensión sobre medicamentos (75,7±13,7) y Plan de Cuidados (64,5±13,2). Pacientes del sexo femenino presentaron mayor promedio en el factor comprensión sobre medicamentos. Los blancos y los residentes en áreas urbanas calificaron mejor el Plan de cuidados. La media más alta se observó para el factor Preferencias aseguradas (84,7±14,3) y la más baja para el factor Plan de cuidados (64,5±13,2). En todos los factores se encontraron diferencias significativas en las variables (paciente quirúrgico, tener artefactos clínicos y no estar hospitalizado por COVID-19). Los pacientes internados hasta cinco días presentaron diferencia estadística en los factores Preparación para el automanejo y Comprensión sobre medicamentos. En los pacientes que no reingresaron dentro de los 30 días posteriores al alta, la preparación para el automanejo fue mejor. Cuanto mejor sea la preparación para el automanejo, menores serán las tasas de reingreso a los 30 días.

**Conclusión:**

en pacientes que viven con enfermedades crónicas, variables sociodemográficas y clínicas están asociadas a la transición del cuidado. Los pacientes que evaluaron mejor la preparación para el automanejo tuvieron menos reingresos dentro de los 30 días.

## Abstract (pt)

**Objetivo:**

avaliar a transição do cuidado na perspectiva de pessoas que vivem com doenças crônicas e identificar sua relação com as características clínicas e sociodemográficas.

**Método:**

estudo transversal, com 487 pacientes que receberam alta de um hospital. Foram utilizados instrumentos de caracterização clínica, sociodemográfica e *Care Transitions Measure*-15, que mensura os fatores Preparo para o autogerenciamento, Preferências asseguradas, Entendimento das medicações e Plano de cuidados. Análise estatística descritiva e inferencial.

**Resultados:**

a transição do cuidado foi satisfatória (76,8±10,4). Média dos fatores: preparo para o autogerenciamento (82,2±10,8), Preferências asseguradas (84,7±14,3), Entendimento das medicações (75,7±13,7) e Plano de Cuidados (64,5±13,2). Pacientes do sexo feminino apresentaram média superior no fator entendimento sobre medicações. Brancos e residentes na zona urbana avaliaram melhor o Plano de cuidados. Observou-se a maior média no fator Preferências asseguradas (84,7±14,3) e a menor no fator Plano de cuidados (64,5±13,2). Em todos os fatores, foram encontradas diferenças significativas nas variáveis (paciente cirúrgico, portar artefatos clínicos e não estar internado por COVID-19). Pacientes internados até cinco dias apresentaram diferença estatística nos fatores Preparação para o autogerenciamento e Entendimento das medicações. Em pacientes que não apresentaram reinternação em 30 dias após a alta, o Preparo para o autogerenciamento foi melhor. Quanto melhor o Preparo para o autogerenciamento, menores são os índices de reinternação em 30 dias.

**Conclusão:**

em pacientes que vivem com doenças crônicas, variáveis sociodemográficas e clínicas estão associadas à transição do cuidado. Pacientes que avaliaram melhor o preparo para autogerenciamento tiveram menos reinternações em 30 dias.

**Highlights:**

**(1)** Brazilian study that evaluated the transition of care of patients with CNCDs.

**(2)** Women had a higher average in the understanding about medications factor.

**(3)** Whites and residents in the urban area better evaluated the care plan.

**(4)** Better preparation for self-management reduces length of stay and readmissions.

**(5)** Better preparation for understanding about medications reduces hospitalization time.

## Introduction

Worldwide, chronic noncommunicable diseases (CNCDs) are responsible for high rates of premature deaths, decreased quality of life (QOL) and increased expenses incurred by families and health systems ^(^ 1 ^)^. In this sense, they demand continuous care from health services, which need organization and integration at the points of the health care network (RAS), so that transition of care (TC) occurs ^(^ 2 ^)^_._ TC is defined as the set of planned actions that aim at the coordination and continuity of patient care, from admission to hospital discharge, as well as the transfer of these individuals between different health services ^(^ 3 ^)^. 

These actions are related to hospital discharge planning and patient and family education for self-care, self-management and medication use, in addition to promoting coordination between services and communication between health professionals for post-discharge follow-up ^(^ 2 ^)^. Hospital-to-home TC and continuity of care in health services are vulnerable processes, especially for people living with chronic diseases, with multiple comorbidities, complicated treatment regimens, or limited caregiver support ^(^ 4 ^)^. 

A study with people living with chronic diseases identified that the lack of planning and involvement of the patient or his family in the education and self-management of his disease and his medications is related to the increase in readmission rates ^(^ 5 ^)^. On the other hand, the literature points out that implementing TC strategies reduces readmissions for preventable causes, increases the QOL of patients and their families, and minimizes the occurrence of adverse events (AE) ^(^ 2 ^)^. 

A systematic review with meta-analysis showed that the implementation of a post-discharge vigilance program reduced 32% of hospital readmissions, while the use of a standardized protocol for enhanced post-surgical recovery significantly reduced the length of stay by up to one and a half days of hospital stay in patients with colorectal cancer ^(^ 6 ^)^. 

Studies to assess the quality of TC, from the perspective of patients and family members during hospitalization, have been carried out using the Care Transitions Measure (CTM), which measures the quality of TC, from hospital discharge to home or between different services, through preparation for self-management, secured preferences, understanding about medications and care plan factors ^(^ 3 ^)^. However, internationally, there are still few studies that evaluate TC from the perspective of people living with CNCDs. Based on a literature review, studies developed in the United Kingdom ^(^ 7 ^)^, the United States ^(^ 8 ^)^, Canada ^(^ 9) ^-^( 10 ^)^ and Sweden ^(^ 11) ^-^( 12 ^)^, were found in international databases, demonstrating that the literature is still incipient. 

In Brazil, two studies that evaluated TC were found: one in the conception of adult and elderly patients with CNCD who were discharged from the emergency service to their homes ^(^ 2) ^,^( 5 ^)^, and another in the conception of patients with digestive system neoplasia that evaluated their TC, as well as identified the factors that influence this process ^(^ 13) ^-^( 14 ^)^. Therefore, there is evidence of a knowledge gap, especially regarding the identification of variables or predictors that can influence the TC, denoting the unprecedented nature of the study. This can contribute to proposing new intervention studies to support strategies for effective transitions, in addition to proposing public policies that discuss this process. According to a review of the literature, studies carried out in Brazil are incipient ^(^ 2) ^,^( 5) ^,^( 14) ^-^( 17 ^)^ with patients who were discharged from hospital admission units, whether clinical or surgical. 

The objective of this study is to evaluate the transition of care from the perspective of people living with chronic diseases and to identify its relation with the clinical and sociodemographic characteristics of the studied population.

## Method

### Study design

This is a cross-sectional study.

### Location or setting in which data collection took place

Clinical and surgical hospitalization units of a general hospital in Rio Grande do Sul, Brazil, which has 126 beds and a hospital occupancy rate between 83% and 87%.

### Period

Data collection was carried out from March to July 2021.

### Population

People living with CNCD.

### Selection criteria

Hospitalized patients with CNCDs, undergoing clinical or surgical treatment, for at least 24 hours, over 18 years old, and who had telephone access after hospital discharge were eligible. Those who were discharged by transfer, who resided in long-stay institutions, as well as those who had a guarded prognosis and did not have the cognitive conditions to answer the questionnaire were excluded, based on the evaluation of the nurse, the first author of this study.

### Sample definition

1112 patients were hospitalized. Of these, 151 did not meet the inclusion criteria, in addition to the occurrence of four refusals, which resulted in 957 eligible patients. Sampling was done for convenience. At the bedside, sociodemographic data were collected, from Monday to Friday, in one shift. Initially, data were collected from 537 patients. They were informed that, after discharge, they would respond to one more instrument, by telephone. There were 50 losses resulting from telephone contact, carried out three times at different times and without success.

### Data collection procedures

Data collection was carried out by a master’s student nurse, first author of the study, at the bedside during hospitalization, through an interview to obtain sociodemographic information (gender, age, address, education, race, marital status). The electronic medical record was accessed for clinical variables (reason for hospitalization, hospitalization unit, length of stay in the unit, number of hospitalizations in the last year, first hospitalization after the diagnosis of COVID-19, continuous use of clinical artifacts such as probes, ostomy bag, drains, among others, after discharge, and chronic diseases). Data regarding hospitalization due to COVID-19 were collected, considering that the collection took place in the pandemic period.

After hospital discharge, between 7 and 30 days, the interviewer applied the CTM-15 ^(^ 3 ^)^ instrument by telephone, validated in Brazil ^(^ 18 ^)^, which measures TC through four factors: preparation for self-management, which values the preparation of the patient and his family for health self-management post-discharge at home; understanding about medications, which refers to the understanding of the patient and his family about the proper use of medications after hospital discharge; secured preferences, which describe the need for patients’ preferences to be considered by the team in decision-making about treatment; care plan, which values the existence of a care plan, consultations or tests to be carried out after discharge ^(^ 3 ^)^. Although there is no cutoff point, the authors consider a score equal to or greater than 70 as a satisfactory TC ^(^ 2 ^)^. Thus, if the overall average is 70, 1 is considered satisfactory. 

After 30 days of collection at the bedside, the data collector returned to the medical record to check if there were readmissions in the period.

### Data treatment and analysis

Data were analyzed using the Statistical Package for the Social Sciences version 25.0, considering a significance level of 5%. Descriptive statistics were applied with absolute and relative distribution and measures of central tendency and variability, with a study of the symmetry of continuous distributions using the Kolmogorov-Smirnov test. The internal consistency of the CTM-15 scale factors was estimated using Cronbach’s alpha coefficient.

For the comparison of each of the CTM-15 factors in relation to the independent variables – treatment, hospitalization due to COVID-19, discharge with clinical artifact, readmission up to 30 days, gender, age group, marital status and place of residence – Student’s t-test was applied for independent groups. The CTM-15 factors considered dependent variables were compared to length of hospital stay and race using the technique of analysis of variance - ANOVA (One Way) - *post hoc* Scheffé (independent groups of very different sizes and/or heterogeneity of variances). 

### Ethical aspects

At the bedside, the participants were invited, the research objectives were explained and they were asked to sign the Free and Informed Consent Form in two copies of equal content. The research was carried out in accordance with Resolution nº 466/2012, approved by the Research Ethics Committee, under Consolidated Opinion 4.479.127/2020.

## Results

487 patients participated in the study. Of these, 205 (42.1%) had a CNCD; 149 (30.6%) two; 90 (18.5%) three; and 42 (8.6%) four or more; 396 (81.3%) of the individuals had been diagnosed with CNCD for more than a year. In addition, 338 (69.4%) were hospitalized due to clinical complications. One hundred and sixty-five patients (34%) were admitted with clinical complications of COVID-19, 146 (30%) with cardiovascular complications, 77 (16%) with endocrine complications and 80 (20%) with other pathologies. Regarding the period of hospitalization, 269 (55.2%) remained hospitalized from 1 to 5 days. It was observed that 341 (70%) of the patients had not been hospitalized in the last year and 209 (42.9%) were undergoing their first hospitalization after the diagnosis of the chronic disease. Thirty-three (6.8%) of the patients had been readmitted within 30 days of discharge. Likewise, 266 (54.6%) were over 60 years old, 256 (52.6%) were female, 292 (53.8%) lived in urban areas, 372 (76.4%) were white, with a mean age of 60.6 years, and 291 (60%) were married or in a stable relationship. As for education, 343 (70.4%) attended an educational institution for up to 8 years.

Regarding the TC assessment, the general mean of the CTM-15 was considered satisfactory (76.8±10.4). When evaluating the averages by factors, the maximum found was in the Secured preferences factor (84.7±14.3), and the minimum in the Care plan factor (64.5±13.2). The internal consistency of the general instrument and by factors was considered satisfactory. In the Care plan dimension, consistency was acceptable, as shown in Table 1. 

**Table 1 -:** Measures of central tendency and variability for the factors of the CTM-15 instrument*. Tenente Portela, RS, Brazil, 2022

CTM-15 Estimates* (n=487)						
**Factors**	**Average**	**Standard deviation**	**Amplitude**		**Median**	αC ^†^
			**Minimum**	**Maximum**		
CMT-15* GENERAL	76.8	10.4	0.00	100	83.3	0.896
Preparation for self-management	82.2	10.8	53.6	100	82.1	0.838
Understanding about medications	75.7	13.7	33.3	100	75.0	0.763
Secured preferences	84.7	14.3	0.0	100	83.3	0.803
Care plan	64.5	13.2	25.0	100	62.6	0.659

*CTM-15 = Care Transitions Measure-15; ^†^αC = Cronbach’s alpha coefficient

The CTM-15 scale was compared with clinical characteristics ( Table 2). When comparing the length of hospitalization of patients, a statistical difference was identified in Preparation for self-management (p<0.003) and Understanding about medications (p<0.003) factors, which denotes higher averages in patients with hospitalization time from 1 to 5 days. There were statistical differences in all factors when comparing the type of surgical or clinical treatment, hospitalization due to COVID-19 or another pathology, and whether the patient was discharged with the presence of clinical artifacts. The highest averages occurred in surgical patients, not hospitalized due to COVID-19, and with the presence of clinical artifacts. 

Furthermore, when comparing patients with readmissions within 30 days after discharge with patients without readmissions, there was a statistical difference between groups (p<0.002) in the Preparation for self-management factor, with a higher average among patients who did not have readmissions (82.6±11.8).

**Table 2 -:** Average and standard deviation of the CTM-15* factors according to the clinical characteristics of patients with CNCD ^†^. Tenente Portela, RS, Brazil, 2022

**Variables**	**Frequency**		CTM-15 Factors* (n=487)							
			**Preparation for self-management**		**Understanding about medications**		**Secured Preferences**		**Care plan**	
	**N** ^‡^	**%**	**Average**	**SD** ^§^	**Average**	**SD** ^§^	**Average**	**SD** ^§^	**Average**	**SD** ^§^
**Length of stay (days)**										
1 to 5	269	55.2	83.5 **A**	10.7	77.2 **A**	14.3	85.8	15.0	64.3	15.7
6 to 14	166	34.1	81.3 **A**	10.7	73.7 **B**	12.9	83.8	13.7	64.0	15.2
15 or more	52	10.7	78.4 **B**	10.1	74.5 **B**	11.8	81.7	12.0	66.8	12.6
P ^||^			**<0.003**		**<0.003**		0.112		0.486	
**Treatment**										
Surgical	149	30.6	85.1	11.1	80.5	13.1	86.7	17.4	73.0	14.7
Clinical	338	69.4	80.9	10.3	73.6	13.4	83.8	12.6	60.7	13.9
P ^¶^			**<0.001**		**<0.001**		**<0.035**		**<0.001**	
**Hospitalized for COVID-19**										
Yes	128	26.3	78.9	9.4	70.1	10.5	80.9	10.2	58.2	12.0
No	358	73.5	83.4	11.0	77.7	14.1	86.0	15.3	66.7	15.7
P ^¶^			**<0.001**		**<0.000**		**<0.001**		**<0.000**	
**Discharge with clinical artifact**										
Yes	219	45	83.8	11.1	78.5	13.7	85.2	16.5	70.1	15.7
No	268	55	80.9	10.4	73.4	13.2	84.2	12.2	59.8	13.1
P ^¶^			**<0.003**		**<0.000**		0.443		**<0.000**	
**Readmission within 30 days**										
Yes	33	6.8	76.8	9.7	75.3	14.0	80.3	19.6	62.9	12.3
No	454	93.2	82.6	11.8	75.7	13.7	85.0	13.8	64.6	15.4
P ^¶^			**<0.002**		0.841		0.069		0.540	

*CTM-15 = Care Transitions Measure-15; ^†^CNCD = Noncommunicable Chronic Disease; ^‡^N = Number; ^§^SD = Standard deviation; ^||^Used the Student’s t-test; ^¶^Used the Analysis of Variance (ANOVA) - *post hoc* Scheffé test, in which averages followed by equal letters do not differ at a significance of 5%

A statistically significant difference was identified in relation to gender, race and place of residence. Female patients had a higher average (77.0±13.5) when compared to the average of male patients (74.3±13.8) in the Understanding about medications factor (p<0.026) of the CTM-15. With regard to race, the Care plan factor (p<0.000) showed a statistical difference when compared to the average of white patients (65.7±15.0) in relation to indigenous patients (54.0±12.1). The variable place of residence had a higher average for the rural area (77.0±14.2) compared to the urban area (74.4±13.0) in the Understanding about medications factor (p<0.034), and a higher average in the urban area (65.9±15.0) concerning to the rural area (63.1±15.1) in the Care plan factor (p<0.047) ( Table 3). 

**Table 3 -:** Average and standard deviation of the CTM-15* factors according to the sociodemographic characteristics of patients with CNCD ^†^. Tenente Portela, RS, Brazil, 2022

**Variables**	**Frequency**		CTM-15 Factors* (n=487)							
			**Preparation for self-management**		**Understanding about medications**		**Secured Preferences**		**Care plan**	
	**N** ^‡^	**%**	**Average**	**SD** ^§^	**Average**	**SD** ^§^	**Average**	**SD** ^§^	**Average**	**SD** ^§^
**Gender**										
Male	256	52.6	82.1	10.7	74.3	13.8	84.5	13.1	64.0	14.6
Female	211	47.4	82.3	10.9	77.0	13.5	84.9	15.3	64.8	15.8
P ^||^			0.841		**<0.026**		0.731		0.550	
**Race**										
Indigenous	40	8.2	81.5	9.1	75.6	13.7	83.1	13.8	54.0 **B**	12.1
White	372	76.4	82.2	11.1	75.8	13.7	84.9	14.7	65.7 **A**	15.0
Black or brown	64	13.1	82.3	9.8	75.4	14.0	83.9	12.4	63.4 **A**	14.0
P ^¶^			0.920		0.974		0.676		**<0.000**	
**Age group**										
18 to 59 years old	221	45.5	81.8	10.1	74.7	13.1	83.9	13.2	64.1	14.8
60 years old or older	266	54.6	82.6	11.3	76.4	14.1	85.3	15.2	64.8	15.6
P ^||^			0.462		0.214		0.270		0.629	
**Marital status**										
Live in union	292	60	82.3	10.8	75.3	14.0	84.6	14.9	63.9	15.2
Does not live in union	195	40	82.0	10.7	76.2	13.1	84.7	13.5	65.4	15.3
P ^||^			0.809		0.625		0.421		0.966	
**Education**										
Up to 8 years	343	70.4	81.2	10.7	75.8	13.3	83.6	14.9	64.0	14.9
Above 8 years	135	27.7	84.6	10.8	75.2	14.6	87.0	12.4	66.1	15.8
P ^||^			0.524		0.282		0.071		0.223	
**Place of residence**										
Urban area	262	53.8	82.0	10.8	74.4	13.0	84.9	13.5	65.9	15.0
Rural area	217	44.6	82.4	10.8	77.0	14.2	84.3	15.3	63.1	15.1
P ^||^			0.724		**<0.034**		0.650		**<0.047**	

*CTM-15 = Care Transitions Measure-15; ^†^CNCD = Noncommunicable Chronic Disease; ^‡^N = Number; ^§^SD = Standard deviation; ^||^Used the Student’s t-test; ^¶^Used the Analysis of Variance (ANOVA) test

## Discussion

The clinical and sociodemographic profile of the investigated population is similar to that presented in another study, of people who reported living with CNCDs ^(^ 19 ^)^. The results of this study show that the TC measured by the CTM-15 in the investigated institution was considered satisfactory. A study carried out in a general hospital in Rio Grande do Sul, Brazil, with patients hospitalized for cancer treatment, identified a similar result ^(^ 13 ^)^. Another study in the same country with patients with CNCDs admitted to the emergency department showed a TC mean of 69.5 ^(^ 2 ^)^. These results indicate that the actions carried out by health professionals at this hospital during hospitalization contribute to a safe discharge and continuity of care. 

To improve the patient’s preparation for the self-management of his health condition in the period after hospital discharge, it is necessary to build some skills for self-management through educational activities, after knowing the patient’s level of understanding about his health situation ^(^ 20) ^-^( 21 ^)^. This contributes a lot to a satisfactory TC, as well as to other evidence-based strategies, such as implementing nurse liaisons in the services, which is not yet a reality in Brazil ^(^ 22 ^)^; use of a validated caregiver assessment tool to systematically evaluate potential gaps in caregiver preparedness and develop a personalized caregiver/family care plan; use of evidence-based teaching and communication methods to optimize caregiver or patient learning and use of technologies to improve nursing care ^(^ 23) ^-^( 24 ^)^. Therefore, the nurse can be the professional responsible for patient education and, through these various strategies, contribute to successful transitions of care ^(^ 20) ^,^( 25 ^)^_._

The percentage of hospital readmissions (6.8%) found in this study is lower than that indicated in an international study, with an average readmission in 30 days of 27.4% ^(^ 26 ^)^. Also, the longer the hospital stay, the lower the perceived quality of returning home ^(^ 12 ^)^. This result constitutes a paradox, since a longer hospital stay means more time in contact with professionals. 

The place of hospitalization is related to the preparation for self-management ^(^ 2 ^)^. A study corroborates this information, pointing out that surgical patients generally have a reduced hospital stay, often with hospitalizations for elective procedures, need for a return visit, in addition to receiving more guidance on home care and, sometimes, educational leaflets distributed by institutions to expand knowledge ^(^ 27 ^)^. 

Patients with a better assessment in the Preparation for self-management factor have lower rates of readmission, which denotes that educating patients facilitates the understanding and management of CNCDs for a longer time after discharge, in addition to helping in their empowerment and understanding of their self-care ^(^ 5 ^)^. 

The better the patient’s planning and preparation for health self-management, the better the patient’s TC experience will be. When comparing patients who were hospitalized for COVID-19 with those who had another cause of hospitalization, there was a statistically significant difference in all CTM-15 factors, and non-COVID-19 patients better evaluated the instructions received and their preparation for discharge. This negative result identified in patients with COVID-19 and patients with CNCDs, possibly evaluated in Brazil for the first time, demonstrates that these patients received less preparation or even were not prepared for hospital discharge, despite being patients with chronic diseases. COVID-19 still raises numerous questions, as it is a disease that requires post-discharge multidisciplinary follow-up due to its possible late complications ^(^ 28 ^)^. 

In our study, patients who returned home with clinical artifacts better evaluated the Preparation for self-management, Understanding about medications and Care plan factors. It is essential that health professionals carry out educational actions throughout the process of preparing for discharge, in order to guarantee patient rehabilitation and, consequently, reduce the occurrence of adverse events (AEs) and returns to services due to preventable causes ^(^ 29 ^)^. 

The ability to understand medication-related information is essential to prevent possible AEs from occurring, especially as there is an ongoing medication need ^(^ 5 ^)^. Among the therapeutic resources for the treatment of CNCDs, medication has become an important component, considering the functional limitations and the risk of complications caused by CNCDs ^(^ 19 ^)^. 

With regard to patients not hospitalized for COVID-19, it is noted that these individuals have a greater understanding about medications, as they are already used to their underlying chronic complications. Patients with COVID-19, in addition to everyday medications, need to deal with specific medications for the treatment of this pathology which, as it is new worldwide, often presents recurrence of some symptoms after returning home ^(^ 28 ^)^. In our study, those who stayed longer in the hospital had better results in terms of understanding about medications, an aspect that is related to the longer time in contact with the health team. 

As for the length of stay, a study shows that the longer the length of stay, the worse the adherence to guidelines, due to the exhaustion related to the long stay in the hospital environment and the higher turnover of companions ^(^ 30 ^)^. In this context, it is important to consider that patients with more severe conditions, with high clinical complexity, tend to need a much longer and more strenuous hospitalization period, both for themselves and for their family members, which can impact their perception of TC quality. 

Also, surgical patients and those who are discharged with a clinical artifact return home with the need for a return visit or follow-up by the basic health units, which facilitates solving doubts regarding drug treatment. This demonstrates the importance and effectiveness of monitoring these individuals through all RAS and knowledge so that they understand and adopt safe drug treatment ^(^ 22 ^)^. 

When the patient’s preferences are included in the decision-making process, adherence to treatment is facilitated, safe conduct at home is increased, and returns to services due to preventable causes are reduced. In the literature, both national ^(^ 2 ^)^ and international ^(^ 12 ^)^, unsatisfactory results were identified in relation to the Secured preferences factor. In contrast, our study demonstrated that this factor was positively evaluated by patients with CNCDs, with the highest average found among the CTM-15 factors. Still, in relation to assured preferences, it is important to highlight that this result is due especially to the perception of patients undergoing surgical treatment and not hospitalized due to COVID-19, who presented higher means with a statistical difference for this factor. According to the literature, assessments of this factor are related to the inclusion of people living with CNCDs and their families in decisions regarding their treatment and post-discharge care, with the integration between staff and patients being fundamental for effective self-care ^(^ 5 ^)^. Therefore, it appears that interventions to ensure patients’ preferences are necessary to improve practices in the discharge process and understanding of their post-discharge care. 

Regarding the Care plan factor, evaluated unsatisfactorily, it is noted that this item has not been prioritized in patient care. This result is especially related to the perception of patients hospitalized due to a clinical condition, without COVID-19, who were discharged without the need for clinical artifacts, residents of rural areas and indigenous people, who presented the lowest means with statistical difference regarding the Care plan factor. This finding may allow the institution where this study was conducted to develop an action plan focused on improving the discharge preparation process and the care plan for this profile of patients. Similar results were found in other studies ^(^ 2) ^,^( 8) ^,^( 12) ^-^( 13 ^)^. It is essential that health services seek processes that enable the establishment of flows and the elaboration of the discharge plan, with the objective that the TCs are adequate between the different levels of assistance and that continuous and articulated care is guaranteed ^(^ 5 ^)^. 

In this sense, the elaboration of the care plan is essential for the continuity of safe care at home and for less need to return to health services due to preventable causes ^(^ 31 ^)^. It must be carried out jointly by the multidisciplinary team and the patient/family member, according to the needs and clinical changes of the individual, health history, prognosis, medications and guidelines on post-discharge follow-up ^(^ 5 ^)^. 

Cultural differences and education can interfere in this process ^(^ 32 ^)^. The indigenous culture links its beliefs, language and systems of knowledge based on traditional medicine to its daily life ^(^ 33 ^)^, in addition to generally having a lower level of education ^(^ 34 ^)^, which may explain the statistical difference identified in this item. 

The elaboration of an individualized care plan, in order to provide continuity of care, is still an incipient practice. A study carried out with a multidisciplinary hospital team points to the need for a discharge plan containing relevant information for treatment and continuity of care ^(^ 13 ^)^. 

Patients using clinical artifacts require greater attention, in view of the educational process for their self-care after hospital discharge, with instructions for the patient and their family members, in order to carry out the care ^(^ 20 ^)^. In this sense, the use of intervention strategies by health services enables more effective TCs, in addition to reducing costs for health services, increasing safety and QL and avoiding hospital readmission rates due to preventable causes ^(^ 5 ^)^. 

Furthermore, it is observed that transitions require an institutional organization, with an established professional team, which must have knowledge of the process and a continuous relationship with both the patient and the care network in the health system, in order to interconnect the discharge process, home care and other services ^(^ 13 ^)^. 

Internationally, nurse liaisons or nurse navigators are being included in the assistance, as professionals who have been demonstrating success in effective TC. Their job is to coordinate post-discharge care, and identify the needs of the patient/family. They also act as educators in transferring information related to discharge planning ^(^ 35) ^-^( 36 ^)^. 

A Brazilian study carried out with nurses shows that there are still gaps to be overcome in user assistance, taking into account the existing fragmentation between services, and indicates some possibilities, such as the organization of protocols that allow: continuity of care, patient transfer by telephone, with appointment scheduling at their reference unit, and use of specific applications, in order to forward the patient to the unit through some technology ^(^ 20 ^)^. 

It was identified that TC requires coordination and communication between the people involved, use of protocols and integration between services in the RAS. It also requires articulation between care practices and national health policies.

This study brings as a contribution the assessment of TC from the perspective of patients with CNCD and its relation with clinical and sociodemographic variables. These results can collaborate with the incorporation of care technologies in order to guarantee the continuity and coordination of the assistance provided to people living with CNCDs within the scope of the RAS.

As a limitation, it is noted that cross-sectional studies present a diagnosis of reality, pointing to associations, but without the possibility of indicating cause and effect. Based on this context, studies with other methodological approaches ^(^ 37 ^)^ are suggested in order to broaden the understanding of these results and test interventions, considering the identified variables and their relationship with the CTM factors. 

## Conclusion

TC, assessed from the perspective of people living with CNCDs admitted to clinical and surgical units, was considered satisfactory in the overall assessment. It was observed that the Preparation for self-management, Understanding about medications and Secured preferences factors presented averages that point to satisfactory results, while the Care plan factor was unsatisfactory. This result is especially related to the perception of patients hospitalized due to their clinical condition, without COVID-19, who were discharged without the need for clinical artifacts, residents of rural areas and indigenous people, who presented the lowest averages with statistical difference regarding the Care plan.

Furthermore, statistical differences were found in the variables: being a surgical patient, being discharged with clinical artifact, and not having been hospitalized due to COVID-19 in all CTM-15 factors. Patients who were not readmitted within 30 days had a better average in the factor Preparation for self-management, which shows the importance of preparing the patient and/or the family member to understand his needs and his care at home, which contributes to the reduction of readmissions and costs in the health system. As for the length of hospital stay, the results are a paradox, as a longer hospital stay is associated with a worse understanding about medications, less satisfaction with secured preferences and a worse perception of self-management. In this context, it is important to consider that patients with more serious conditions, with high clinical complexity, tend to need a much longer and more strenuous period of hospitalization, both for the patient and for the family members, which can impact their perception of the quality of the TC. It can also be considered that the clinical or surgical hospitalization, discharge with clinical artifact and hospitalization or not due to COVID-19 variables are also mediating this association found.

The results contribute to identifying aspects that need to be enhanced in care to support the development of strategies to improve TC.
